# A bright organic NIR-II nanofluorophore for three-dimensional imaging into biological tissues

**DOI:** 10.1038/s41467-018-03505-4

**Published:** 2018-03-21

**Authors:** Hao Wan, Jingying Yue, Shoujun Zhu, Takaaki Uno, Xiaodong Zhang, Qinglai Yang, Kuai Yu, Guosong Hong, Junying Wang, Lulin Li, Zhuoran Ma, Hongpeng Gao, Yeteng Zhong, Jessica Su, Alexander L. Antaris, Yan Xia, Jian Luo, Yongye Liang, Hongjie Dai

**Affiliations:** 1 Department of Materials Science and Engineering, South University of Science and Technology of China, 518055 Shenzhen, China; 2 Department of Chemistry, Stanford University, Stanford, CA 94305 USA; 3 JSR Corporation, Advanced Materials Research Laboratories, 100 Kawajiri-Cho, Yokkaichi, Mie 5108552 Japan; 4 Department of Physics and Tianjin Key Laboratory of Low Dimensional Materials Physics and Preparing Technology, School of Sciences, Tianjin University, 300350 Tianjin, China; 5 Department of Chemistry, Tsinghua University, 100084 Beijing, China; 6 Palo Alto Veterans Institute for Research, VA Palo Alto Health Care System, Palo Alto, CA 94304 USA; 7 Department of Neurology and Neurological Sciences, School of Medicine, Stanford University, Stanford, CA 94305 USA

## Abstract

Fluorescence imaging of biological systems in the second near-infrared (NIR-II, 1000–1700 nm) window has shown promise of high spatial resolution, low background, and deep tissue penetration owing to low autofluorescence and suppressed scattering of long wavelength photons. Here we develop a bright organic nanofluorophore (named p-FE) for high-performance biological imaging in the NIR-II window. The bright NIR-II >1100 nm fluorescence emission from p-FE affords non-invasive in vivo tracking of blood flow in mouse brain vessels. Excitingly, p-FE enables one-photon based, three-dimensional (3D) confocal imaging of vasculatures in fixed mouse brain tissue with a layer-by-layer imaging depth up to ~1.3 mm and sub-10 µm high spatial resolution. We also perform in vivo two-color fluorescence imaging in the NIR-II window by utilizing p-FE as a vasculature imaging agent emitting between 1100 and 1300 nm and single-walled carbon nanotubes (CNTs) emitting above 1500 nm to highlight tumors in mice.

## Abstract (web summary)

Imaging in the second near-infrared window has attracted attention due to superior penetration depth and low signal interference. Here, the authors describe a new organic nano fluorophore with high quantum yield and demonstrate its use for in vivo imaging.

## Introduction

Compared to conventional fluorescence imaging in the visible and near-infrared spectrum window of 400–900 nm, a newly developed fluorescence imaging approach that extends fluorescence wavelength to the second near-infrared window (NIR-II, 1000–1700 nm) has received much interest^1, 2^. NIR-II fluorescence imaging in vivo can benefit from diminished tissue autofluorescence and reduced photon scattering without high level of light absorption, allowing deep tissue penetration and high-clarity fluorescence imaging into a living body^3, 4^. Thus far, most NIR-II fluorophores based on organic molecules have suffered from low quantum yield (QY) in aqueous solutions due to strong interactions with water molecules and the resulting dominance of non-radiative decay pathways^5–7^. This has limited the capability of in vivo NIR-II imaging in reaching real-time tracking at high speeds and frame rates, and deciphering 3D structures of live tissues in a layer-by-layer fashion by developing *z*-resolved imaging techniques in the 1000–1700 nm range. Up to now, in most studies^5–12^ NIR-II imaging is limited to 2D projected epi-fluorescence imaging without the capability of gleaning 3D tissue structures deeply, partly due to the lack of sufficiently bright biocompatible fluorophores (QY around 0.3–2%)^5–12^.

Here, we employ an organic NIR-II dye (FE) exhibiting a high QY in organic solvents such as toluene. To retain the high QY in aqueous solutions for biological imaging, we encapsulate the dye in the hydrophobic interior of an amphiphilic polymer, poly(styrene-co-chloromethyl styrene)-graft-poly(ethylene glycol) (PS-*g*-PEG), to produce a bright and biocompatible NIR-II nanofluorophore (referred to p-FE, hydrodynamic size ~12 nm). The PS backbone forms a hydrophobic core mimicking the toluene environment to encapsulate the dye and prevents its aggregation and fluorescence quenching, while the hydrophilic PEG chains impart p-FE with aqueous solubility and biocompatibility. The QY of p-FE in aqueous environment is estimated up to ~16.5% based on the IR26 reference fluorophore (QY_IR26_: ~0.5%)^13^, which is among the brightest organic molecule based NIR-II fluorophores for biological imaging. With this bright nanofluorophore, non-invasive ultra-fast in vivo NIR-II imaging of blood flow in the brain vessels of mice with a short exposure time of 2 ms is realized. The bright nanofluorophore enables 3D confocal layer-by-layer imaging of formalin fixed mouse brain tissue to resolve vessels with apparent widths of ~5–7 µm in diameter at imaging depth up to ~1.3 mm in the NIR-II window, which is the deepest 3D fluorescence microscopy of brain tissues using the one-photon fluorescence technique. p-FE is also an excellent imaging agent for blood vasculatures with an ultra-long blood circulation (half-life time ~16 h), and a novel probe for tumors with an impressive T/NT signal ratio of ~12 through the EPR effect. Lastly, we succeed in using two colors in the 1000–1700 nm range for simultaneous in vivo fluorescence imaging of a tumor and blood vasculatures surrounding the tumor using carbon nanotubes (CNTs) and p-FE, respectively.

## Results

### Synthesis and characterization of nanofluorophore p-FE

**Fig. 1:**
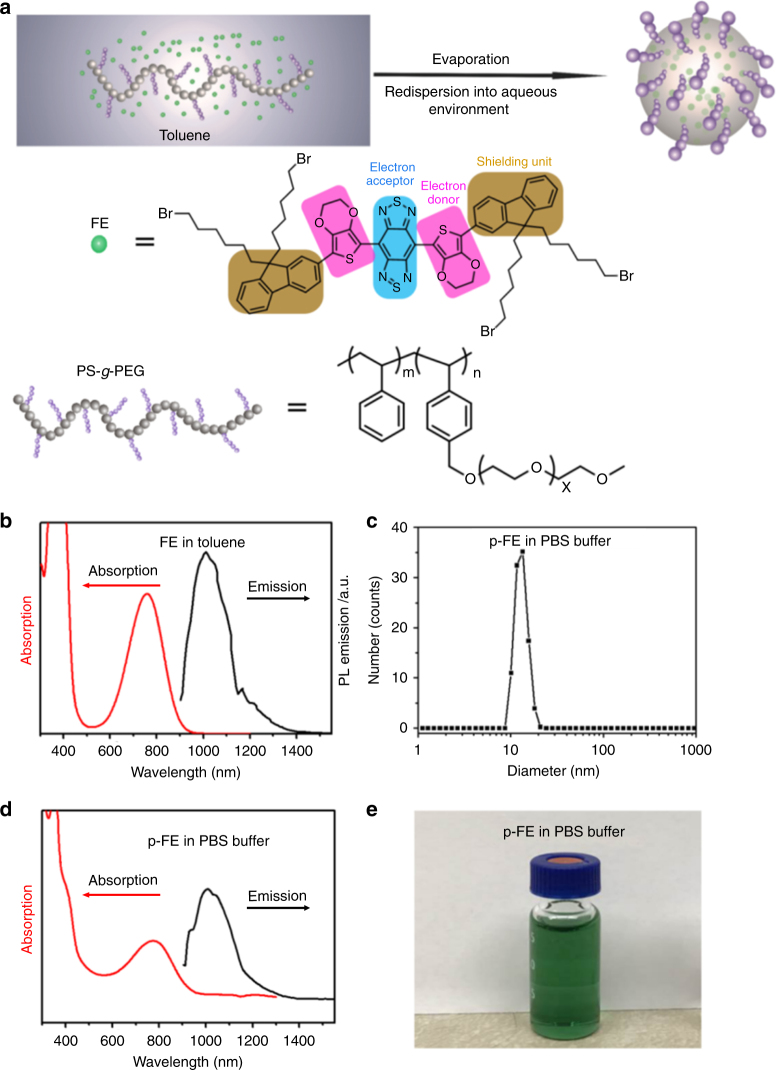
Synthesis and characterization of p-FE. **a** Scheme of p-FE synthesis and chemical structures of FE and the PS-*g*-PEG polymer. **b** Absorption and emission spectra (excited by an 808 nm laser) of FE in toluene. **c** Dynamic light scattering (DLS) analysis of p-FE in PBS buffer. **d** Absorption and emission spectra (excited by an 808 nm laser) of p-FE in PBS buffer. **e** Photo of p-FE dispersed in PBS buffer. Mw of PS-*g*-PEG is around 23000 g moL^−1^

We synthesized a hydrophobic NIR-II organic dye (FE) with shielding unit-donor-acceptor-donor-shielding unit (S-D-A-D-S) structure constructed by benzobisthiadiazole (BBTD) as the acceptor (A), 3, 4-ethylenedioxy thiophene (EDOT) as the donor (D), and dialkyl fluorene as the shield unit (S) (Fig. 1a and Supplementary Fig. 1). The FE dye exhibited high QY in toluene, attributed to the EDOT and dialkyl fluorene moieties in the molecule. Specifically, the bulky EDOT affords a larger backbone distortion and a less delocalized lowest unoccupied molecular orbital (LUMO), while the alkyl chains on fluorene stretch out of the plane of conjugated backbone, preventing the backbone from aggregation and interactions with other molecules^5^. FE exhibited an absorption peak around 760 nm (mass extinction coefficient ~13.0 L g^−1^ cm^−1^ in toluene) and broad tunable emission range from 1000 to 1400 nm with a peak of ~1010 nm in the NIR-II window under the excitation of an 808 nm laser (Fig. 1b).

To obtain a bright NIR-II fluorophore for biological imaging and retain the high QY of the FE dye, we encapsulated FE in an amphiphilic polymer matrix to make it biocompatible and bright (Fig. 1a). As shown previously^14, 15^, amphiphilic polymers can be used to trap hydrophobic fluorophores in the core, while the hydrophilic groups of the polymer impart aqueous solubility of the complex. We performed living reversible addition-fragmentation chain-transfer polymerization (RAFT) to synthesize a PS-*g*-PEG amphiphilic structure with controlled molecular weight and polydispersity to encapsulate FE via evaporation induced assembly process (See Methods and Supplementary Fig. 2). Specifically, the PS backbone formed a hydrophobic core mimicking the toluene environment to retain the high QY of FE and the PEG side chains off the backbone imparted the complex with aqueous solubility and biocompatibility, thus affording a bright NIR-II fluorophore (p-FE) highly soluble in aqueous solutions.

Dynamic light scattering (DLS) analysis revealed that p-FE exhibited a hydrodynamic diameter centered at ~12 nm, forming a nanofluorophore suitable for in vivo biological imaging (Fig. 1c). The small size of the nanofluorophores was also confirmed by transmission electron microscope (TEM) and atomic force microscope (AFM) (Supplementary Fig. 3). p-FE demonstrated superior solubility in PBS, FBS, and cell media (The concentration of p-FE can reach 1000 mg mL^−1^ or even higher when dispersed in PBS, FBS, and cell media, which is enough for biological applications). The p-FE nanofluorophore in solution also showed high photostability (Supplementary Fig. 4), with an absorption peak at ~774 nm and emission from 1000 to 1350 nm (peak ~1010 nm) under an 808 nm laser excitation (Fig. 1d). The relative QY of p-FE in aqueous environment was estimated to be ~16.5% based on the IR26 reference fluorophore (QY_IR26_: ~0.5%)^13^, about 40 times higher than that of single-walled carbon nanotubes (CNTs) used for the first mouse NIR-II imaging^16^. The stable physicochemical and optical properties of p-FE were confirmed in warm serum (37 °C fetal bovine serum, FBS) over a period of one week (Supplementary Fig. 5). Note that the QY and size of p-FE were tunable by varying the loading amount of FE (Supplementary Fig. 6). To balance the brightness and a suitable hydrodynamic size in the nanometer scale, we tuned p-FE nanofluorophore to ~12 nm in size with a QY of ~16.5% for subsequent biological imaging experiments.

### Real time fast NIR-II imaging

We carried out a series of non-invasive in vivo imaging to glean the capabilities of p-FE for NIR-II imaging of mice. High spatial resolution owed to reduced scattering of fluorescence emission in the NIR-II window by tissues allowed us to clearly observe blood vessels inside mouse brain through intact scalp skin and skull after intravenous injection of p-FE. We succeeded in dynamic imaging and tracking of blood flow in the cerebral-vasculatures of mice using a short exposure time of 2 ms under 808 nm excitation with a safe illumination power of ~140 mW cm^−2^ within a video-imaging time duration of <600 s (actual imaging time needed was much shorter). To achieve a safe laser power, the laser beam in our study was expanded from 0.0013 to 35 cm^2^ and excitation filters were used (850 and 1000 nm short-pass filters). Note that the actual illumination power received by the mice was only ~70 mW cm^−2^ after passing through the excitation filter set, much lower than the safe laser power limit of ~329 mW cm^−2^ at 808 nm (according to the International Commission on Non-ionizing Radiation Protection^17^, for laser wavelength (*λ*) between 700–1050 nm, safe laser power limit can be calculated as: EL = 0.2×10^[0.002(*λ*−700)]^ W cm^−2^ if the exposure duration *t* = 10−30000 s, which leads to EL at 808 nm is ~329 mW cm^−2^). Immediately after tail vein injection of p-FE, video-rate imaging of blood flow of brain vessels in a mouse with a frame rate of 47.6 frames per second (FPS) could be achieved (see real-time, >30 FPS imaging of blood flow, Supplementary Movie 1). The frame rate was limited by the InGaAs camera employed in this work since the camera overhead time was 19 ms between frames. With the p-FE nanofluorophore, one could expect an upper limit of 500 FPS corresponding to the 2 ms exposure time.

**Fig. 2:**
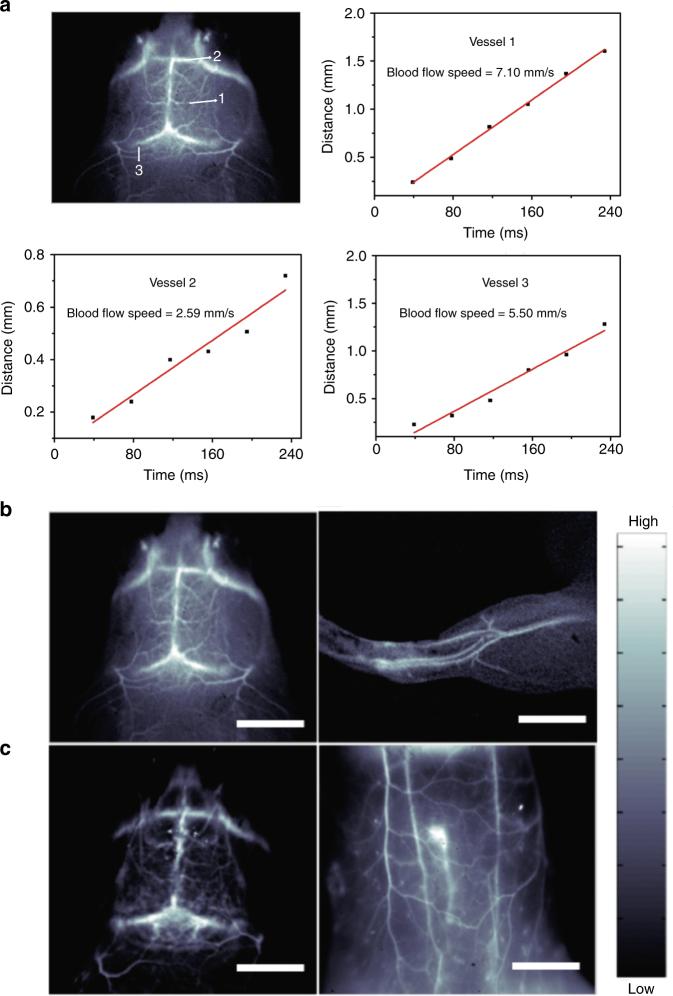
Non-invasive real-time in vivo fluorescence imaging of mice intravenously injected with p-FE. **a** Ultra-fast imaging of blood flow in brain vessels of a mouse injected with p-FE with low exposure time of 2 ms through collection of fluorescence emitting above 1100 nm. **b**, **c** High-magnification fluorescence imaging of brain, hindlimb, and belly of the mouse through collection of fluorescence emitting above 1100 nm with low exposure time of 2 ms (**b**) and above 1300 nm with exposure time of 20 ms (**c**). Scale bars in Fig. 2b represent 6 mm and scale bars in Fig. 2c represent 6 and 10 mm, respectively

The high temporal and spatial resolution afforded by p-FE allowed us to map out the fast-moving blood flows in the brain vessels of mice in an entirely non-invasive manner without the need of craniotomy. The blood flow velocity in different vessels was quantified by plotting the distance traveled by the blood flow front imaged in the NIR-II window immediately post tail-vein injection (Fig. 2a).

Static imaging with exposure time of 1 and 2 ms produced crisp images of mouse brain and hindlimb vasculatures by detecting emission from the circulating p-FE above 1000 and 1100 nm, respectively (Fig. 2b and Supplementary Fig. 7). Furthermore, despite the peak emission of p-FE being ~1010 nm, we were able to utilize its substantial emission tail extended above 1300 nm for imaging with low exposure time of ~20 ms in the 1300−1400 nm (Fig. 2c), NIR-IIa window^18, 19^ known to further suppress photon scattering for enhancing image clarity and signal to background ratios over imaging in the ~1000 nm region. Utilization of such emission tail for long wavelength imaging to benefit from low tissue scattering was shown in our earlier imaging of traumatic brain injury^7^. More recently, emission tails of conventional NIR-I dyes extending into the >1000 nm NIR-II window were reported by us (Supplementary Fig. 14 in ref. ^20^) and others^21, 22^.

### One-photon 3D confocal imaging in the NIR-II window

Thus far, imaging in the NIR-II window has been mainly done in wide-field epi-fluorescence mode with 3D structural information projected to a 2D plane. In contrast, confocal imaging collects signal from a small probe volume at the focus while rejecting out-of-focus signal, allowing for 3D imaging in a layer-by-layer fashion by scanning the laser focal point in *x-y-z* directions. However, a requirement for confocal imaging is highly bright fluorophores in order to perform deep 3D imaging with a safe laser intensity to achieve sufficiently high scanning speed/short exposure time pixel by pixel in a reasonable time frame compatible with biological samples. Notably, conventional one-photon confocal imaging in the visible or NIR-I (700–950 nm) window in the past has been limited to an imaging depth <360 µm^23, 24^ by light scattering.

We built an one-photon confocal imaging system in the NIR-II window for biological imaging using p-FE, achieving imaging depth of ~1 mm with high spatial resolution (sub-10 µm). We adopted lenses that were anti-reflection-coated in the NIR-II region, an IR objective with 3.2 mm long working distance, a 785 nm NIR-II continuous wave excitation laser, an 850 nm long-pass dichroic, and a photomultiplier (PMT) sensitive in the 950–1700 nm range to construct the confocal setup that differed from any previous confocal setup. Scanning was done with a sample placed on a 3D piezo-electric stage moving along *x, y*, and *z* directions for large area (~10 × 10 cm) at a relatively slow speed. Fast scanning and imaging were done in the *x-y* plane in areas of 200 × 200 µm using galvo mirrors, with *z*-scanning done by moving the piezo-electric stage. The fluorescence signals were detected by the PMT point by point for image construction. A small diameter pinhole was adopted to exclude out-of-focus fluorescence and restrict images to one narrow focal plane.

**Fig. 3:**
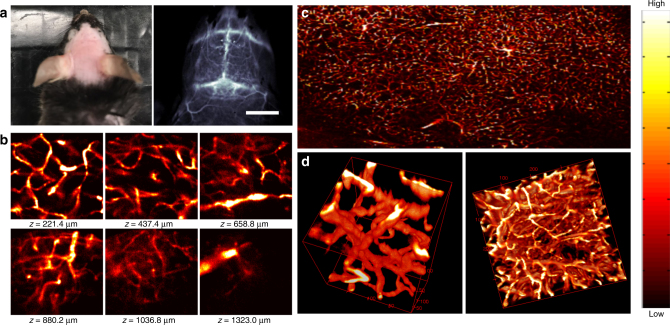
Ex vivo confocal imaging of brain vasculatures of a mouse injected with p-FE. **a** Photo and wide-field NIR-II epi-fluorescence imaging of brain in a mouse injected with p-FE (808 nm excitation, emission >1200 nm) with exposure time of 5 ms. **b**–**d** Ex vivo confocal imaging of brain in a mouse injected with p-FE (785 nm excitation, emission >1100 nm, laser power ~30 mW, PMT voltage ~500 V). **b** Small area (200 × 200 µm, *x* × *y*, step size: 1 µm) and **c** Large area (3000 µm × 2000 µm, *x* × *y*, step size: 1 µm). The deepest area could reach ~1350 µm. **d** 3D reconstruction of vasculatures in brain: small area (left side, 200 µm × 200 µm × 200 µm, *x* × *y* × *z*, step size: 1 µm along *x, y*, and *z* directions, galvo mirror scanning, scanning speed: 2 s/frame) and large area (right side, 400 µm × 400 µm × 400 µm, x × y × z, step size: 2 µm along *x* and *y* directions, 2.7 µm along *z* direction, stage scanning, scanning speed 7.5 min frame^−1^). Scale bar represents 6 mm

Owing to the high QY of p-FE, we succeeded in using confocal imaging ex vivo to decipher 3D vasculatures in fixed mouse brain tissue immersed in glycerol through collection of fluorescence emitting above 1100 nm under the excitation of a tightly focused 785 nm laser (see Method for details). Small vessels with apparent widths of ~5–7 µm in diameter at depth up to ~1.3 mm in the brain tissue were resolved. This was the deepest 3D fluorescence microscopy among one-photon techniques (see Fig. 3, 3D reconstructed image animation, Supplementary Movie 2 and layer-by-layer image animation, Supplementary Movie 3).

For in vivo NIR-II confocal imaging, we circumvented the motion problem caused by mouse breathing by imaging the mouse hindlimb where breathing related motion was the smallest. After deletion of frames with breathing artifacts and 3D reconstruction, we observed clear vasculatures in the mouse hindlimb under the confocal imaging mode, demonstrating 3D one-photon confocal imaging in vivo in live mice (Supplementary Fig. 8).

### In vivo two-color tumor imaging in the 1000–1700 nm window

**Fig. 4:**
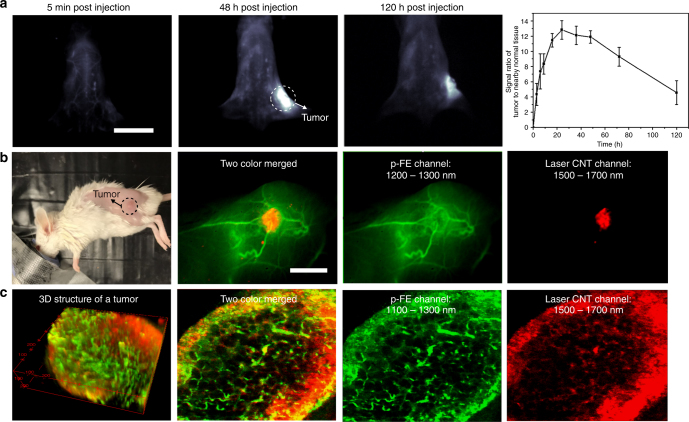
Two-color fluorescence imaging of a tumor in the NIR-II window. **a** Wide-field fluorescence imaging of a mouse inoculated with a 4T1 tumor through collection of fluorescence emitting from p-FE above 1300 nm with exposure time of 5 ms and variation of T/NT signal ratio as a function of time (*n* = 3). **b** High-magnification fluorescence imaging of a mouse inoculated with a 4T1 tumor using two colors in the NIR-II window. **c** Confocal imaging of a tumor using two colors in the NIR-II window. 740 µm  × 740 µm × 220 µm area, step size: 2 µm along *x* and *y* directions, 5.4 µm along *z* direction. Laser power ~30 mW, PMT voltage: 500 V for p-FE channel and 600 V for laser CNT channel, scanning speed 15 min/frame. Pin hole: 150 µm for p-FE and 300 µm for laser CNT channel. Wavelength range: 1100–1300 nm for p-FE channel and 1500–1700 nm for laser CNT channel. Scale bars in Figs 1a and 2b represent 2 cm and 6 mm, respectively. Error bars correspond to standard deviation

Cancer imaging is key to diagnosis and therapeutic interventions and has been pursued by a wide range of modalities including fluorescence techniques^3^. Fluorophores with high tumor uptake can highlight a tumor and discriminate cancerous parts from normal tissues, beneficial for various clinical applications (e.g., imaging guided surgery, drug delivery, mechanism study)^25–28^. Owing to the ~12 nm hydrodynamic size and long blood circulation (half-life time of ~16 h, see next section), p-FE is expected to exhibit high tumor accumulation through the EPR effect^25, 29, 30^. To evaluate tumor imaging capability of p-FE, mice inoculated with the xenograft 4T1 murine breast tumor (size around 33 mm^3^) were injected with p-FE through the tail vein, followed by NIR-II imaging and monitoring of tumors overtime. As p-FE circulated over time the tumor became brighter and brighter, reaching a peak at 48 h post injection (p.i.). The T/NT signal ratio reached as high as ~12 (Fig. 4a), much higher than previous non-targeted NIR-I ICG fluorophores and antibody-targeted fluorophores^31, 32^, suggesting the potential of p-FE for tumor imaging based on the EPR effect.

Because of the high accumulation and high QY of p-FE, the tumor could be still clearly discriminated from other normal tissues by collecting fluorescence emitting above 1200 and 1300 nm with short exposure time of 1 and 5 ms, respectively (Supplementary Fig. 9). To further identify the impressive passive tumor targeting ability of p-FE through the EPR effect, the mouse inoculated with the 4T1 tumor was sacrificed after intravenous injection of p-FE and the tumor and other major organs were taken out for imaging. Ex vivo imaging also found p-FE richly accumulated inside the tumor among major organs, consistent with in vivo imaging results (Supplementary Fig. 10).

Next we performed in vivo two-color fluorescence imaging in the NIR-II window, motivated by the potential of probing and differentiating multiple components or molecular targets in deep tissues. By utilizing the fluorescence of p-FE emitting in 1100–1300 nm for one color to highlight vasculatures and that of laser-ablation produced CNTs emitting in 1500–1700 nm for another color to highlight the tumor, we succeeded in profiling a mouse tumor model in vivo (Fig. 4b). After first intravenous injection of biocompatible laser-ablation produced CNTs^33^ into a mouse bearing a 4T1 tumor and circulation overnight, CNTs accumulated within the tumor through the EPR effect and were mostly cleared out of circulation. p-FE was then injected into blood circulation to highlight blood vessels around and in the tumor and was immediately imaged in a fluorescent channel different from the CNTs under the same 808 nm laser illumination. After overlaying these two colors, we found vessels around and in the tumor were abundant, feeding to aggressive tumor growth as reported in the literature (Fig. 4b)^34, 35^.

In addition to in vivo wide-field fluorescence imaging, ex vivo 3D layer-by-layer confocal imaging was used to glean the internal structures of the tumor shortly after injection of p-FE and sacrificing the mouse at 1 h p.i. (Fig. 4c). We observed that due to the leaky tumor vasculatures^36^, overnight circulation of CNTs led to extravasation of some CNTs out of the vessels into the surrounding tumor tissue, with the vessels highlighted by p-FE that were still mostly circulating in the blood vessels (see Method for more details). Therefore, p-FE and CNTs predominantly highlighted the vessels and the tumor, respectively, in two colors (see Fig. 4c, 3D reconstructed two-color image animation, Supplementary Movie 4, and Supplementary Fig. 11). It was also observed that the fluorescence emitting from CNTs and p-FE were much higher in the outer region of the tumor, suggesting the outer region of the tumor were much richer in tumor vasculatures and allowed higher CNT extravasation than that in the tumor interior.

### In vivo pharmacokinetics and biocompatibility of p-FE

**Fig. 5:**
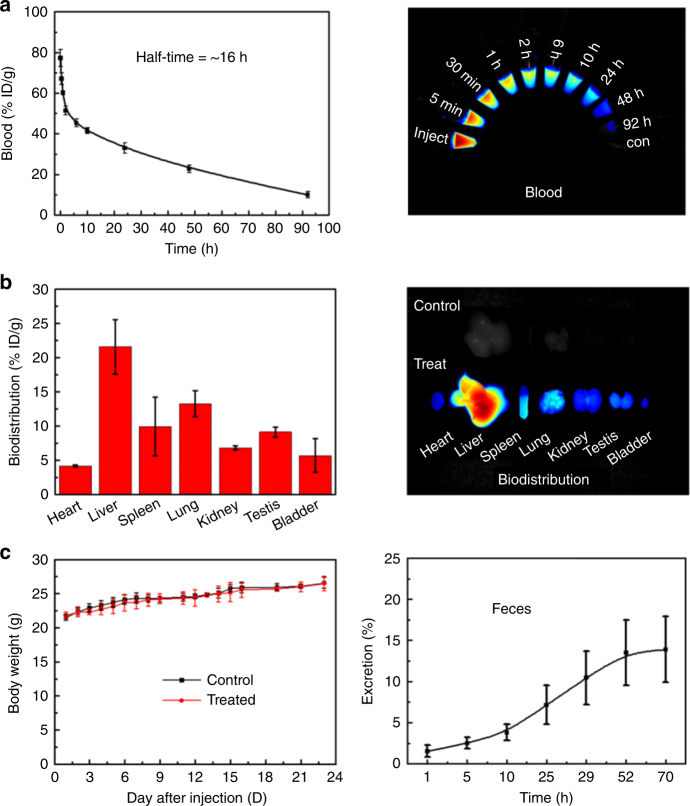
Evaluation of pharmacokinetics and biocompatibility of p-FE (*n* = 3 mice/group). **a** Time course of blood concentration in 92 h. Fluorescence signal decreased from 77.31 to 9.91%, with a long blood circulation half-life time of ~16 h and corresponding fluorescence images of blood samples. **b** Biodistribution of main organs of p-FE-treated mice after 24 h post injection. **c** Body weight of p-FE-treated mice (*n* = 6 mice/ group) over a period of time of 23 days and the excretion behavior of p-FE. Error bars correspond to standard deviation

To investigate the pharmacokinetics of p-FE in vivo, C57 mice were intravenously injected with p-FE at the same dose as used for imaging. As shown in Fig. 5a, the percentage of p-FE in blood was 77.31% ID g^−1^ at 5 min p.i. and decreased to 9.91% ID g^−1^ at 92 h p.i., showing a prolonged blood half-life time of ~16 h (the method for the determination of blood half-life time was based on previous works^6, 37^), which was 8–16 times longer than previously reported organic NIR-II fluorophores such as, CH1055, IR-E1, and IR-FEP^5–7^. Biodistribution at 24 h p.i. showed that liver was the dominant organ for accumulation of p-FE with amount of 21.6% ID g^−1^, followed by accumulation of 13.2% ID g^−1^ in the lung (Fig. 5b). The other organs including heart, spleen, kidney, testis, and bladder showed low uptake. p-FE was gradually excreted via feces overtime, suggesting the hepatobiliary clearance pathway (Fig. 5c).

In vivo toxicity of p-FE was examined at 1 and 23 days p.i. Body weight showed no significant difference between control and p-FE-treated groups (Fig. 5c). The hematology indicators did not show significant fluctuation during the monitoring period (Supplementary Fig. 12). Serum biochemistry results showed slight transient decrease of albumin and albumin/globulin at 1 day p.i., but these serum indicators recovered to healthy level at 23 days p.i. (Supplementary Fig. 13). Two important liver indicators, aspartate transaminase (AST), and alanine transaminase (ALT) also showed no significant fluctuation, indicating no detectable liver injury during the period. Pathology analysis by H&E staining indicated no morphological changes in all the tested organs (Supplementary Fig. 14). In addition, neurobehavioral tests using RotaRod and SmartCages^38^ revealed no motor deficits in p-FE-treated groups. Postmortem analysis of brain tissues of p-FE-treated mice showed no gross changes of brain morphology (Nissl/cresyl violet staining) and no signs of neuroinflammation (CD68 immunostaining), indicating there was no brain injury (Supplementary Fig. 15). Collectively, all the evaluations demonstrated the high biocompatibility of p-FE.

## Discussion

In summary, we developed a bright organic NIR-II nanofluorophore (p-FE) with a small hydrodynamic size of ~12 nm through encapsulation of hydrophobic dye FE into an amphiphilic matrix. The high brightness (QY: ~16.5%) of the organic nanofluorophore allowed non-invasive in vivo imaging using short exposure time down to 2 ms by detecting fluorescence emitting above 1100 nm under 808 nm excitation, enabling real-time tracking of blood flow at a high frame rate of 47.6 FPS in mouse brain non-invasively. Confocal imaging of intact biological tissues was achieved with one-photon imaging depth of ~1 mm with sub-10 µm high spatial resolution, demonstrating the deepest 3D fluorescence microscopy for one-photon fluorescence techniques. p-FE was an excellent vasculature imaging agent with bright NIR-II fluorescence and long blood circulation (half-life time ~16 h), which favored the passive targeting of a 4T1 tumor with an impressive tumor/normal tissue signal ratio of ~12 through the EPR effect. In vivo two-color imaging of a tumor in the NIR-II window was realized by extracting fluorescence of p-FE emitting in 1100–1300 nm and fluorescence of laser CNT emitting in 1500–1700 nm, opening exciting potentials for monitoring and understanding tumor behaviors in vivo and in situ using fluorescence microscopy.

Combining reduced tissue scattering and high brightness of the p-FE nanofluorophore, its superior aqueous stability, long blood circulation, and excellent biocompatibility, we demonstrated several pre-clinical applications of advanced NIR-II imaging. Such imaging could facilitate real-time monitoring and visualization of vascular abnormalities and 3D reconstruction to map out vascular structures toward diagnosis and imaging guided therapy. In the future, p-FE can be imparted with new functions and bio-specificity for further research. For example, hydrophobic drugs can also be loaded into p-FE to obtain a theranostic nanoplatform and functional groups can be imparted at the terminal of PEG chains on p-FE for modification of targeting moieties to achieve molecular imaging of specific targets in vivo.

## Methods

### Synthesis of PS-*g*-PEG

PS backbone was synthesized using RAFT of styrene and 4-(chloromethyl)styrene. Typically, styrene (0.34 g, 3.26 mmol), 4-(chloromethyl) styrene (1.00 g, 6.55 mmol), chain transfer agent 4-cyano-4-(phenylcarbonothioylthio) pentanoic acid (0.028 g, 0.10 mmol), and initiator AIBN (0.004 g, 0.02 mmol) were mixed in a 25 mL Schlenk flask, and the solution was degassed by three freeze-pump-thaw cycles. The mixture was then heated under nitrogen at 90 °C for 20 h. The polymerization was stopped by cooling the reaction flask in liquid nitrogen. The resulting copolymer was isolated as a pink powder (0.60 g) after dissolving in DCM and precipitating into 20-fold MeOH for three times (The PS copolymer: *M*_n_ = 7900 g/mol, *Đ = *1.07). To graft mPEG onto the PS copolymer backbone, copolymer (0.05 g), mPEG (*M*_n_~1000) (0.35 g, 0.35 mmol, one equivalent to chloromethyl groups on the PS copolymer), and NaOH (0.020 g, 0.5 mmol) were added into anhydrous THF (1.5 mL). The mixture was stirred for 18 h at room temperature under nitrogen. The reaction mixture was filtered to remove the generated salt and residual base, and the filtrate was evaporated to dryness. The crude graft polymer was then dialyzed against DI water for three days and lyophilized to give a white powder (0.42 g). PS-*g*-PEG: *M*_n_ = 23000 g/mol, *Đ = *1.26.

### Formation of a bright NIR-II nanofluorophore p-FE

One hundred microliters of FE dye (synthesized through our previous protocol^5^) dissolved in toluene (OD 808 = 3.74) and 10 mg PS-*g*-PEG was mixed homogenously under sonication for 90 s at room temperature. Then the toluene was evaporated at 37 °C overnight. All substances were reconstituted with 200 µL PBS buffer under sonication for 10 min. The mixture was centrifuged at 8500 r.p.m. for 30 min to remove precipitates. The amount of remaining p-FE was calculated based on the adsorption at 774 nm measured by UV-Vis-NIR spectroscopy.

### Mouse handling

Mouse handling was approved by Stanford University’s administrative panel on Laboratory Animal Care. All experiments were performed in accordance with the National Institutes of Health Guide for the Care and Use of Laboratory Animals. C57 and BALB/c mice were purchased from Charles River. Bedding, nesting material, food, and water were provided. Eight-week-old BALB/c mice were shaved and inoculated with ~2 million 4T1 cells on the right side for tumor growth. All experiments relating to mice were replicated within 3–6 times. The sample sizes of mice were selected based on previously reported studies. Mice were randomly selected from cages for all experiments. No blinding was performed. All relevant data are available from authors.

### **In vivo** wide-field fluorescence imaging in the NIR-II window

Unless otherwise noted, all NIR-II fluorescence images were recorded using a 2D liquid-nitrogen cooled InGaAs camera (Princeton Instruments 2D OMA-V, USA) under the excitation of an 808 nm laser (RPMC Lasers, USA). To realize ultra-fast imaging of blood flow in brain vessels, a C57 mouse was placed on an imaging stage positioned in front of the InGaAs camera. An 808 nm fiber-coupled diode laser (RPMC Lasers, USA) was used as the excitation source and an excitation filter set (850 and 1000 nm short-pass filter) was used to filter the excitation light. The video-rate imaging was done with low exposure time of 2 ms (47.6 FPS due to 19 ms overhead time during readout) using p-FE for injection with an OD of 6.5 (808 nm) at 200 µL volume and fluorescence emitting from p-FE was directed into the InGaAs camera through emission filter sets (900 and 1100 nm long-pass filters). For imaging the mouse above 1000 nm, 900 and 1000 nm long-pass filters, and low exposure time of 1 ms were used. Fluorescence images were plotted and analyzed using MATLAB software. For mouse hindlimb or whole-body imaging, the same laser, imaging stage, excitation filter set, and camera were used, except for the imaging area. For evaluation of the tumor accumulation capacity of p-FE, a BALB/c mouse inoculated with a 4T1 tumor was injected with p-FE. The tumor was monitored as a function of time through collection of fluorescence emitting above 1200 and 1300 nm with low exposure time of 1 and 5 ms, respectively.

### **Ex vivo** and **in vivo** confocal imaging in the NIR-II window

To probe internal vasculatures of brain in a mouse, p-FE dispersed in PBS buffer with OD of 6.5 (808 nm) was injected intravenously into a C57 mouse at the volume of 200 µL. The mouse was sacrificed at 1 h p.i. of p-FE and the brain or tumor tissue was taken out and fixed with 10% neutral buffered formalin at 4 °C and preserved in glycerol at 4 °C before confocal imaging. The sample was mounted on coverglass slide and immersed in glycerol for confocal imaging with the objective lens in air. The imaging depth was corrected considering the refractive index difference of air and sample (calculated correction factor is 1.8162 in consideration that refractive index of air and brain tissue is 1.0003 and 1.3526, respectively, and the numerical aperture of our objective is 0.8).

For in vivo confocal imaging of a hindlimb, a C57 mouse injected with p-FE was anesthetized during imaging and the excitation laser was focused on the hindlimb (see more details in the Supplementary Information). Since the QY of CNTs is much lower than that of p-FE, for two-color imaging of a tumor in the NIR-II window, we injected a high dose of CNTs and used five times longer exposure time for CNT imaging. Also, for two-color 3D confocal imaging, we used a larger size pinhole for CNTs (~300 µm) than that (~150 µm) for p-FE. This allowed more fluorescence emission from CNTs to be collected but it resulted in a lower *z*-resolution. Also, the PMT voltage used for CNTs (600 V) was higher than that of p-FE (500 V). Laser CNTs were firstly injected into a BALB/c mouse inoculated with a 4T1 breast tumor with OD of 15 (808 nm) at the volume of 250 µL. After circulating overnight to let laser CNT accumulate within the tumor, p-FE was then injected to light up vessels around and in the tumor. The mouse was sacrificed at 1 h p.i. of p-FE and the tumor was taken out and fixed with formalin for confocal imaging. To record images, we used a home-built galvo mirror scanning confocal setup. We used lenses that were anti-reflection-coated in the NIR-II region, an IR objective with 3.2 mm long working distance, a 785 nm NIR-II continuous wave excitation laser, an 850 nm long-pass dichroic and a PMT sensitive in 950–1700 nm to construct the confocal setup, which distinguish our NIR-II confocal setup from any conventional confocal setup. The 100× objective (Olympus, oil immersion, NA 0.8, USA) focused excitation laser to a tiny spot with a few micrometer diameter onto the sample, and the fluorescence went through 850 nm long-pass dichroic and emission filters to the PMT detector. For p-FE channel, 1100 nm long-pass filter and 1300 nm short-pass filter were used as emission filters and 150 µm pinhole was used. For laser CNT channel, 1500 nm long-pass filter was used as the emission filter and 300 µm pinhole was used. Signal was detected with a NIR PMT (Hamamatsu H12397-75, Japan). For galvo scanning pictures, frame rate was 2 s/frame, and pixel rate was 50 µs/pixel. For stage scanning pictures, the scanning speed is limited by how fast the stage moves. We can simply increase the scanning speed by increasing the speed that the stage moves.

### In vivo pharmacokinetics and biocompatibility of p-FE

C57 mice were used for pharmacokinetics, excretion, and biodistribution studies (*n* = 3 mice/group). All mice were intravenously injected with p-FE at the same dose for imaging. The method for the determination of blood half-life time was based on our previous works^6, 37^. In detail, at time points of 0.08, 0.5, 1, 2, 6, 10, 24, 48, and 92 h, 50 µL blood was collected using a capillary tube stuck into the corner of one eye, and then diluted with EDTA-Na_2_ solution (40 mg mL^−1^). The concentration of p-FE in blood was estimated by the fluorescent intensity using an in vivo fluorescence imaging system (Caliper Inc., USA) in 830–940 nm, under the excitation of 733 nm. The percentage of p-FE in blood was calculated by subtracting the background from the blood of healthy mice. Biodistribution was performed after 1 and 23 days intravenous injection. Main organs including heart, liver, spleen, lung, kidney, testis, and bladder were collected for fluorescence imaging. In this study we used organ homogenate instead of intact tissues to precisely quantify the biodistribution of p-FE. The homogenate solution was obtained by complete ultrasonication of tissues (IKA, T18, ULTRA-TURRAX) in saline. Afterward, all samples were placed for hours to facilitate the residue sedimentation, followed by twice centrifugation. The biodistribution was quantified by the fluorescence signal in organ homogenate. For excretion experiment, the feces were collected for four days. Distilled water (four times as weight) was added into each sample, mixing by ultrasound for 1 h, and then the feces were milled into homogenate. All processed samples were imaged by fluorescence imaging system. The amount of excretion was calculated as follow:

$${\mathrm{Excretion}} = \frac{{{\sum}_1^{n} {V_{n} \times I_{n}} }}{{V \times I}}$$

where *V* is the injected volume and *I* the fluorescence intensity.

C57 mice (7–8 weeks) were employed for toxicity experiments and randomly divided into four groups (*n = *6 mice/group), which were control and treated groups for 1 day, control and treated groups for 23 days. After intravenous injection of p-FE at the same dose for imaging, mice were weighed every day and scarified at 1 and 23 days for hematology, serum biochemistry, and pathology studies. Blood of each mouse was collected and analyzed by a blood cell counter (Mindray, BC-2800 Vet). The rest of blood samples were left under 4 °C overnight, then centrifuged at 6000 r.p.m. for 8 min. The supernatant serum was separated for biochemistry testing (HT 7150). For pathology evaluation, all mice were sacrificed by anesthesia and exsanguination. Main organs including heart, liver, spleen, lung, kidney, testis, and bladder were fixed in 10% formalin for 48 h and made into paraffin section. All slides were disposed according to a standard H&E staining protocol and checked by an optical microscope (Olympus).

### Neurobehavior testing and neurotoxicity assessment

RotaRod testing and SmartCage monitoring were performed at five days after p-FE injections according to published protocols^38^. Mice were then perfused, and brains were dissected, fixed, and sectioned at 40 μm with a sliding microtome 2010 (Leica, Allendale, NJ, USA). The sections were stained with cresyl violet and with an antibody against CD68 (for microgliosis and neuroinflammation)^37^.

### Statistical analyses

Results were presented as mean ± SD. Statistical significance was determined by a two-tailed Student’s *t* test. A *P*-value of less than 0.05 was considered significant.

### Data availability

All relevant data are available from the authors.

## Electronic supplementary material

Supplementary Information(PDF 1945 kb)

Description of Additional Supplementary Files(DOCX 14 kb)

Supplementary Movie 1

Supplementary Movie 2

Supplementary Movie 3

Supplementary Movie 4
