# Supplementary material for: A bright organic NIR-II nanofluorophore for three-dimensional imaging into biological tissues
Source: Nat Commun. 2018 Mar 21;9:1171. doi: 10.1038/s41467-018-03505-4 (PMC5862886; doi:10.1038/s41467-018-03505-4)
Supplement: Supplementary file 1 — Supplementary Information(PDF 1945 kb) [file 41467_2018_3505_MOESM1_ESM.pdf]

A Bright Organic NIR-II Nanofluorophore for Three-Dimensional Imaging into Biological Tissues

Wan et al

A Bright Organic NIR-II Nanofluorophore for Three-Dimensional Imaging into Biological Tissues

Hao Wan[1,2,8], Jingying Yue[2,8], Shoujun Zhu[2,8], Takaaki Uno[2,4,8], Xiaodong Zhang[3,8], Qinglai Yang[1,5,8], Kuai Yu[2], Guosong Hong[2], Junying Wang[3], Lulin Li[6], Zhuoran Ma[2], Hongpeng Gao[2], Yeteng Zhong[2], Jessica Su[2], Alexander L. Antaris[2], Yan Xia[2], Jian Luo[6,7], Yongye Liang[1] and Hongjie Dai[2]**

[1] Department of Materials Science and Engineering, South University of Science and Technology of China, Shenzhen 518055, China.

[2] Department of Chemistry, Stanford University, Stanford, California 94305, USA.

[3] Department of Physics and Tianjin Key Laboratory of Low Dimensional Materials Physics and Preparing Technology, School of Sciences, Tianjin University, Tianjin 300350, China.

[4] JSR Corporation, Advanced Materials Research Laboratories, 100 Kawajiri-Cho, Yokkaichi, Mie 5108552, Japan.

[5] Department of Chemistry, Tsinghua University, Beijing 100084, China.

[6] Palo Alto Veterans Institute for Research, VA Palo Alto Health Care System, Palo Alto, California 94304, USA.

[7] Department of Neurology and Neurological Sciences, School of Medicine, Stanford University, Stanford, California 94305, USA.

[8] These authors contribute equally to this work

Correspondence should be addressed to: hdai@stanford.edu, liangyy@sustc.edu.cn

Materials. Unless otherwise noted, all reagents were obtained commercially and used without further purification. 4-(Chloromethyl)styrene (St-Cl:＞90%, TCI, USA) and styrene (St:＞99%, Sigma-Aldrich, USA) were destabilized by passing through a basic alumina column. 4-Cyano-4-(phenylcarbonothioylthio)pentanoic acid (CTPA) was purchased from Strem Chemicals Inc, USA. Azoisobutyronitrile (AIBN, Sigma-Aldrich, USA) was purified by twice re-crystallizing in methanol. Sodium hydroxyl (NaOH), anhydrous tetrahydrofuran (THF, Sigma-Aldrich, USA), and polyethylene glycol monomethyl ether 1000 (m-PEG1000, TCI, USA) were used as received. Laser CNT was obtained according to our previous protocol. Tetrahydrofuran (THF), toluene, and dimethyl formamide (DMF) used for reactions were purified by solvent purification system (Innovative Technology, Inc. UK) before using. All air and moisture sensitive reactions were carried out in flame dried glassware under a nitrogen atmosphere.

***Ex vivo* and *in vivo* confocal imaging.** In detail, a mouse injected with p-FE was sacrificed less than 2 h post injection, and the whole brain was taken out and fixed with 10% neutral buffered formalin at 4℃and preserved in glycerol at 4℃before confocal imaging. The sample was mounted on coverglass slide and immersed in glycerol for confocal imaging with the objective lens in air. The imaging depth was corrected considering the refractive index difference of air and sample (calculated correction factor is 1.8162 in consideration that refractive index of air and brain tissue is 1.0003 and 1.3526, respectively, and the numerical aperture of our objective is 0.8). In the galvo mirror scanning mode, the stage with the brain tissue only moved along z direction and two galvo mirrors scanned the laser beam in x and y directions with the area of 200 μm * 200 μm to obtain 2D images. Larger area scanning done by moving the stage (stage mode) along x and y directions with step sizes of 1 μm showed the overall distribution of vessels in 3 mm * 2 mm area. After reconstruction of all frames scanned under galvo and stage modes continuous 3D vasculatures of the mouse brain were obtained. Such bright fluorophore also can make *in vivo* confocal imaging in the NIR-II window come true. Considering that the motion caused by breath will affect

the position of the mouse, thereby making images in the z direction look discontinuous, we chose to focus on a hindlimb which was less affected by breath as the object for *in vivo* confocal imaging. By adoption of galvo mirror imaging mode and step size of 2 μm along z direction, a hindlimb of the mouse was scanned layer by layer. After deletion of frames with breathing defects and 3D reconstruction, clear vasculatures showed.

Characterization. 1H NMR spectra were recorded in $CDCl_3$ using a Varian Inova 300 spectrometer, USA. Chemical shifts were reported in parts per million relative to $CDCl_3$ (δ7.27). Gel permeation chromatography (GPC) measurements were performed in THF on two PLgel 10 m mixed-BLS columns (Agilent Technologies, USA) connected in series with a DAWN 8+ multi-angle laser light scattering (MALLS, USA) detector and an Optilab TrEX differential refractometer (Wyatt Technology, USA). No calibration standards were used, and dn/dc values were obtained for each injection by assuming 100% mass elution from the columns. Dynamic light scattering (DLS) characterization was performed on Zetasizer (Malvern, UK) at 25 ^{0}C using PBS buffer as the dispersing agent to determine the hydrodynamic diameter of samples. The method we adopted for analysis was the cumulant method. The UV-Vis-NIR absorption spectra were measured on Carry 5000 spectrophotometer (Agilent Technologies, USA). The NIR fluorescence spectrum was taken using an in-house-built NIR spectroscopy set-up. The 808 nm excitation laser (RPMC Lasers, USA) was allowed to pass through the solution sample in a 10-mm-path-length cuvette (Starna Cells, USA), and the emission was collected in a transmission geometry. The emission light was directed into a spectrometer (Acton SP2300i, USA) equipped with a liquid-nitrogen-cooled InGaAs linear array detector (Princeton OMA-V, USA) for detection between 900-1800 nm (QY was calculated by collecting fluorescence emitting in this range). Spectra were corrected post-collection to account for the sensitivity of the detector and extinction feature of the filter.

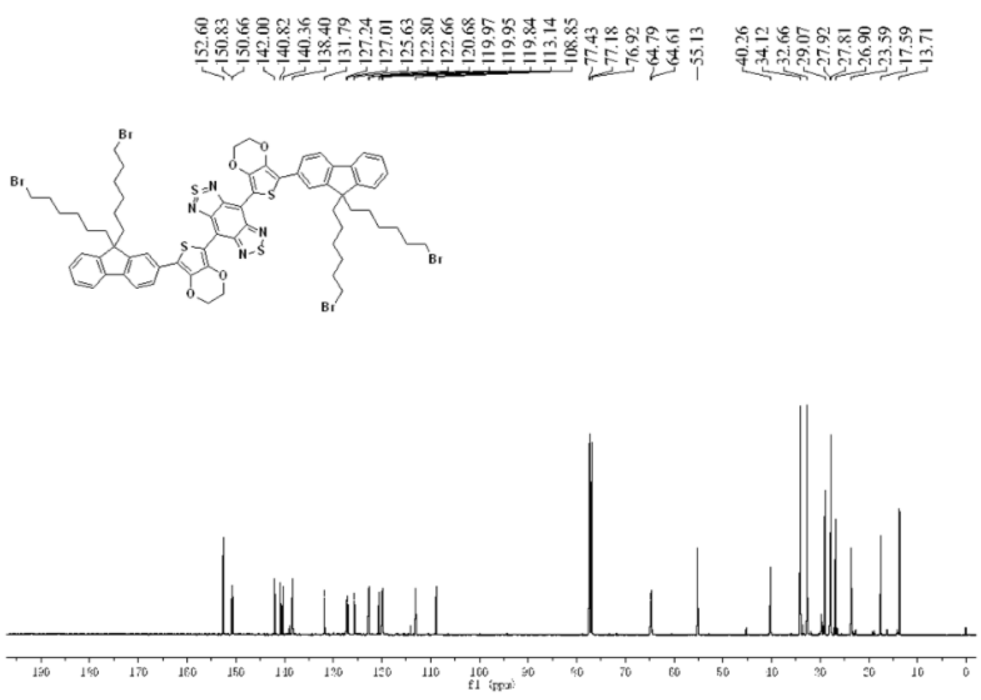

Supplementary Figure 1. NMR analysis of FE.

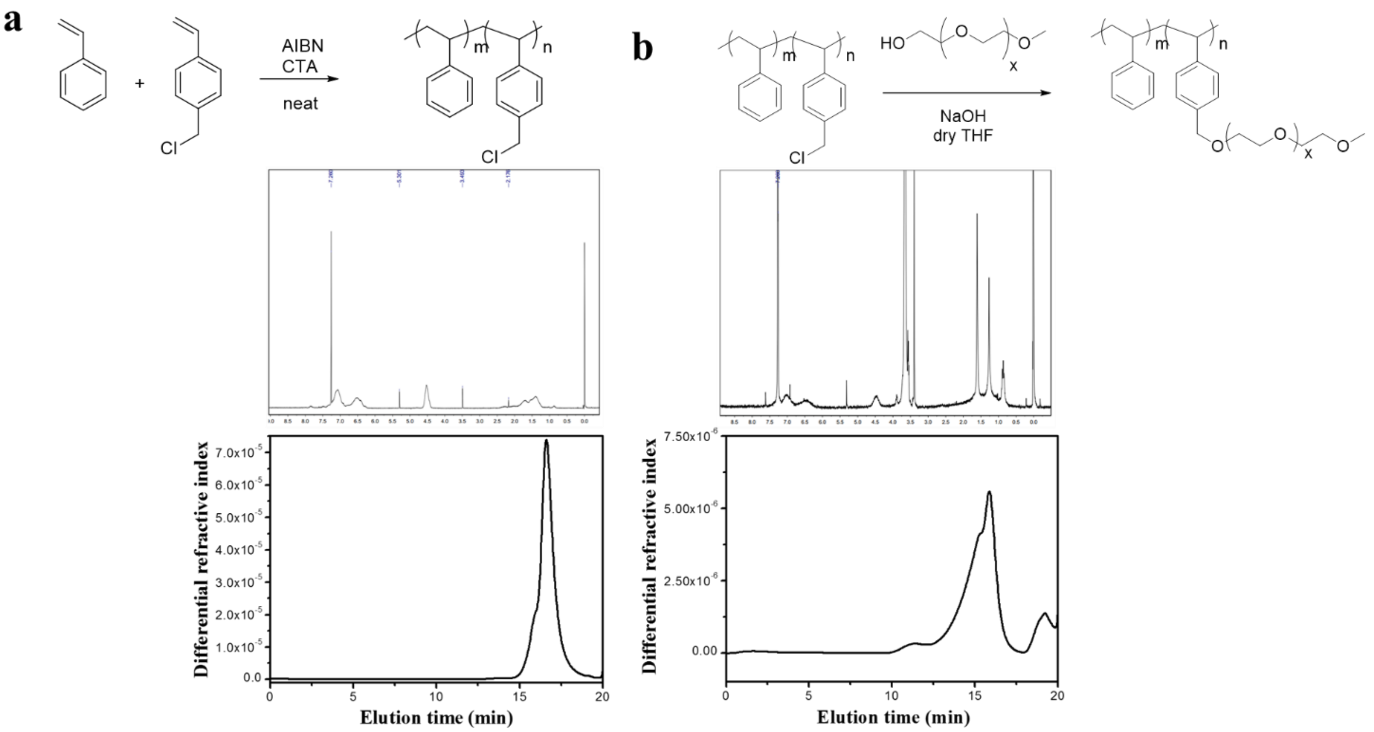

Supplementary Figure 2. NMR and GPC characterization. a, Precursor PS-PSCl and **b,** PS-*g*-PEG.

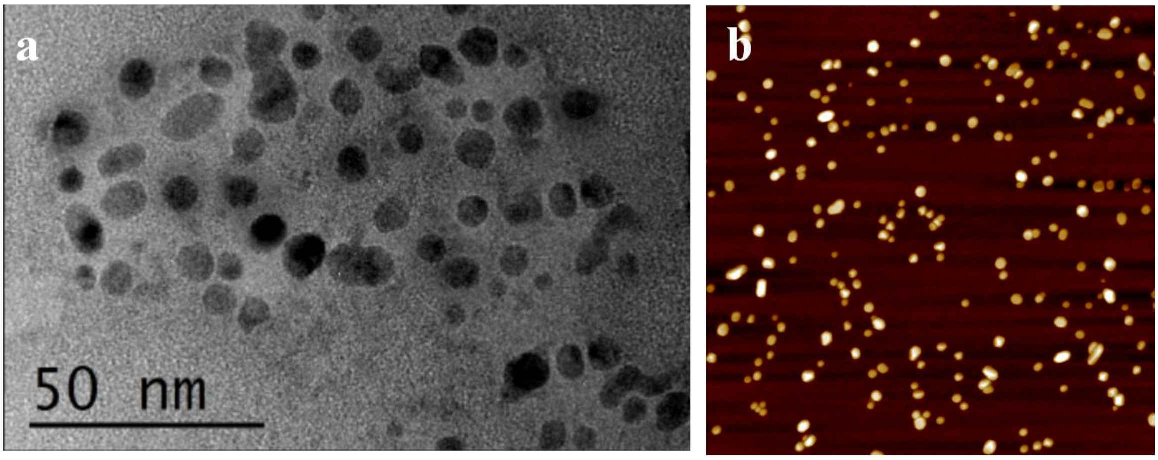

Supplementary Figure 3. a, TEM and **b,** AFM analyses of p-FE (scanning area: 5 μm * 5 μm)

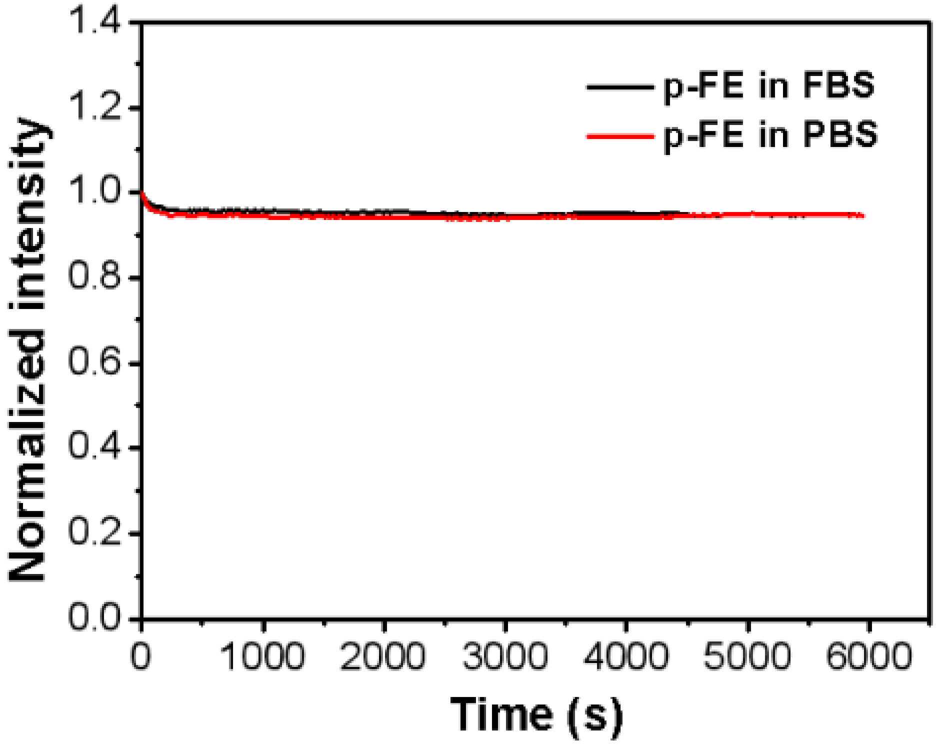

Supplementary Figure 4. Evaluation of the photo-stability of p-FE. Under continuous irradiation of an 808 nm laser (70 mW/cm^{-2}) for ~ 6000 s, the brightness of p-FE decayed only ~ 5% and ~ 6% in PBS and FBS, respectively.

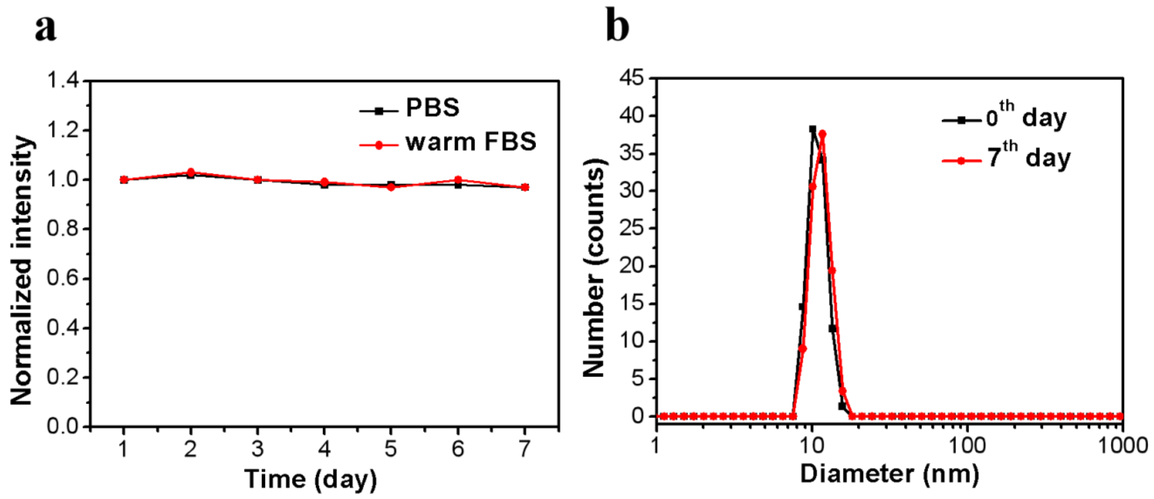

Supplementary Figure 5. a, Fluorescence emission intensity of p-FE in PBS solution and 37 ºC warm fetal bovine serum (FBS) solution as a function of days. **b,** Long-term stability of p-FE in 37 ºC warm FBS solution reflected by the hydrodynamic size.

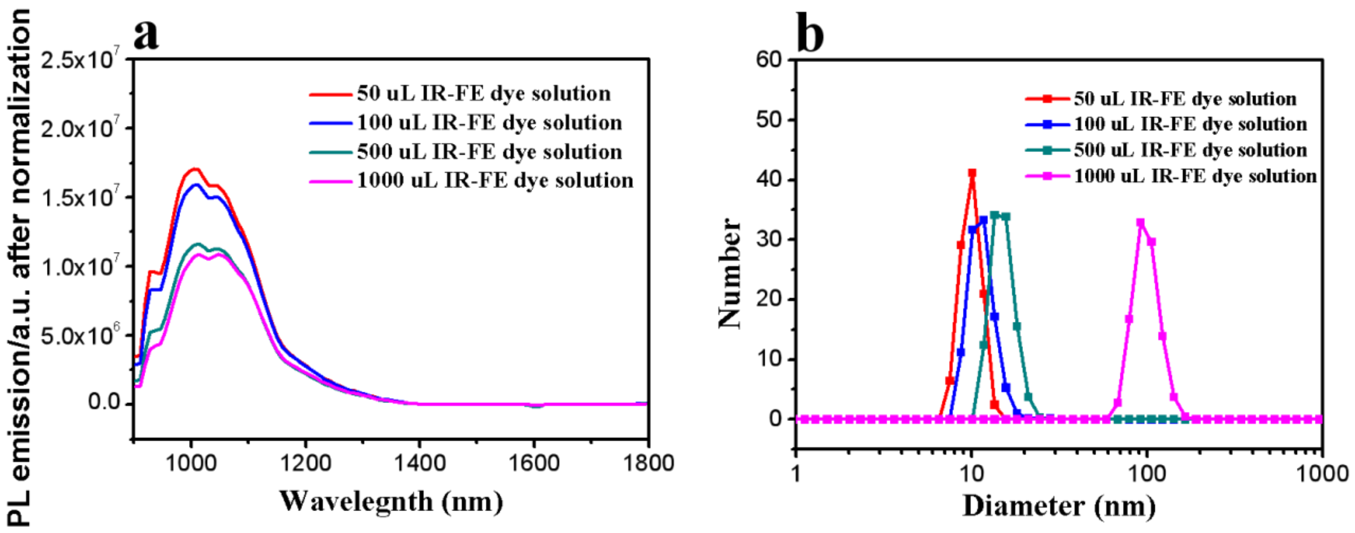

Supplementary Figure 6. a, Size and **b,** QY of p-FE with different loading amount of FE (amount of PS-g-PEG was fixed).

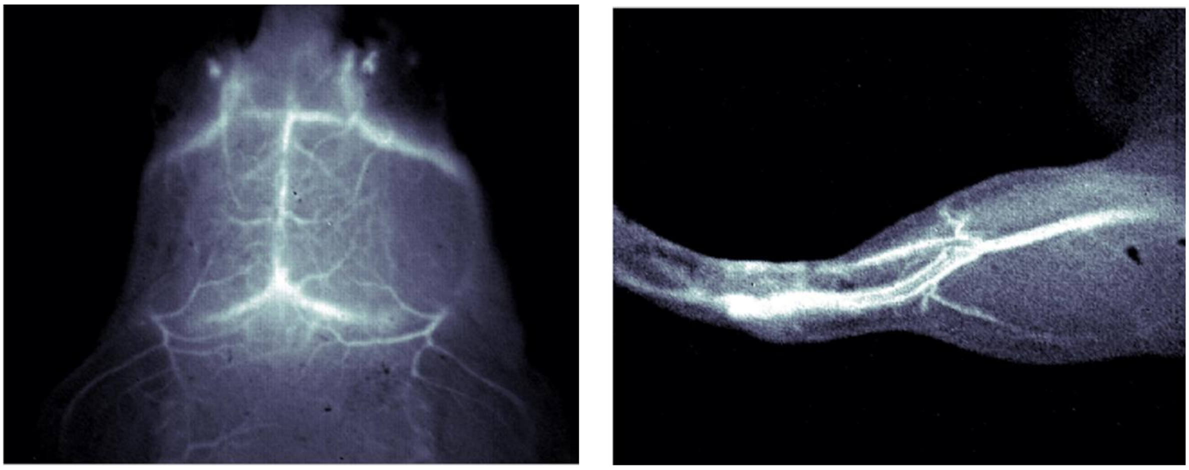

Supplementary Figure 7. High-magnification fluorescence imaging of the brain and hindlimb of a mouse injected with p-FE through collection of fluorescence emitting above 1000 nm with low exposure time of 1 ms.

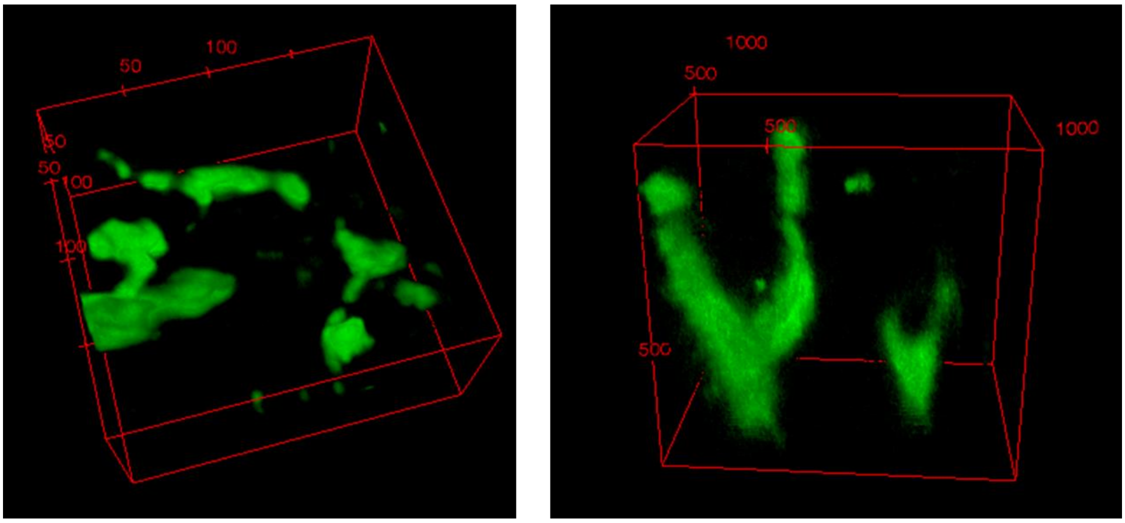

Supplementary Figure 8. *In vivo* confocal imaging of a hindlimb of a mouse injected with p-FE through collection of fluorescence emitting above 1100 nm.

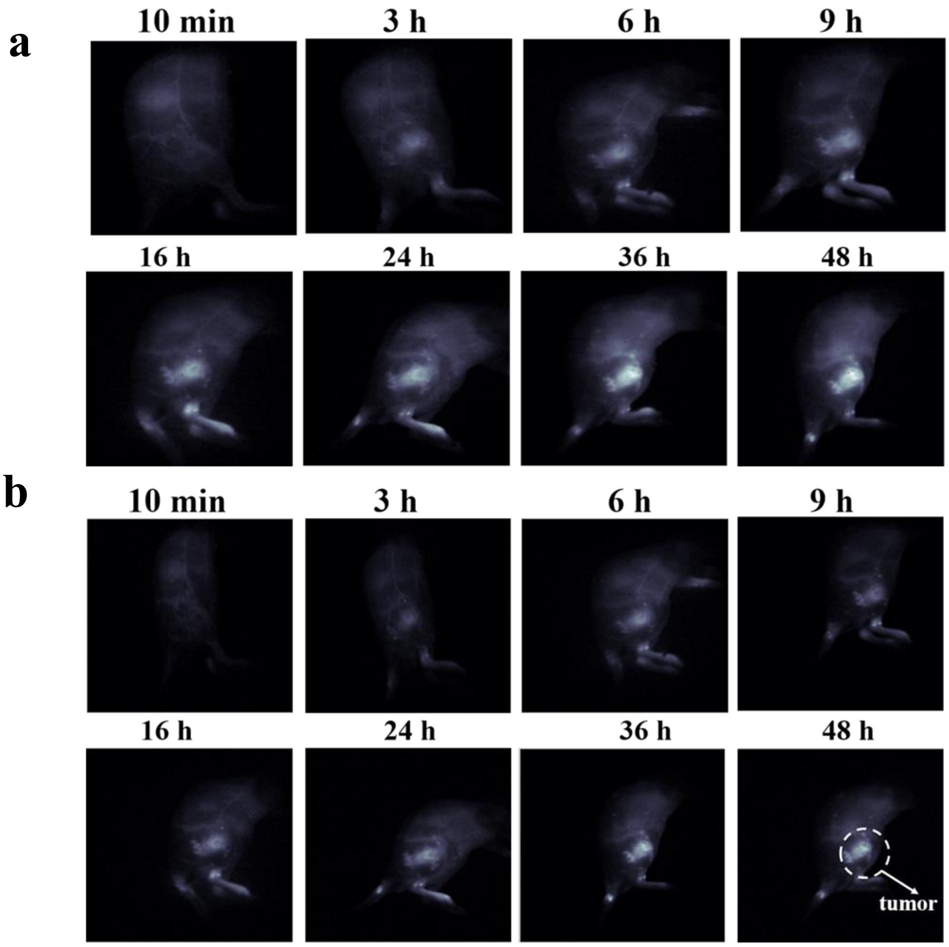

Supplementary Figure 9. Wide-field fluorescence imaging of a mouse inoculated with a 4T1 tumor as a function of time. Image after intravenous injection of p-FE through collection of fluorescence emitting above **a,** 1200 nm with exposure time of 1 ms and **b,** above 1300 nm with exposure time of 5 ms.

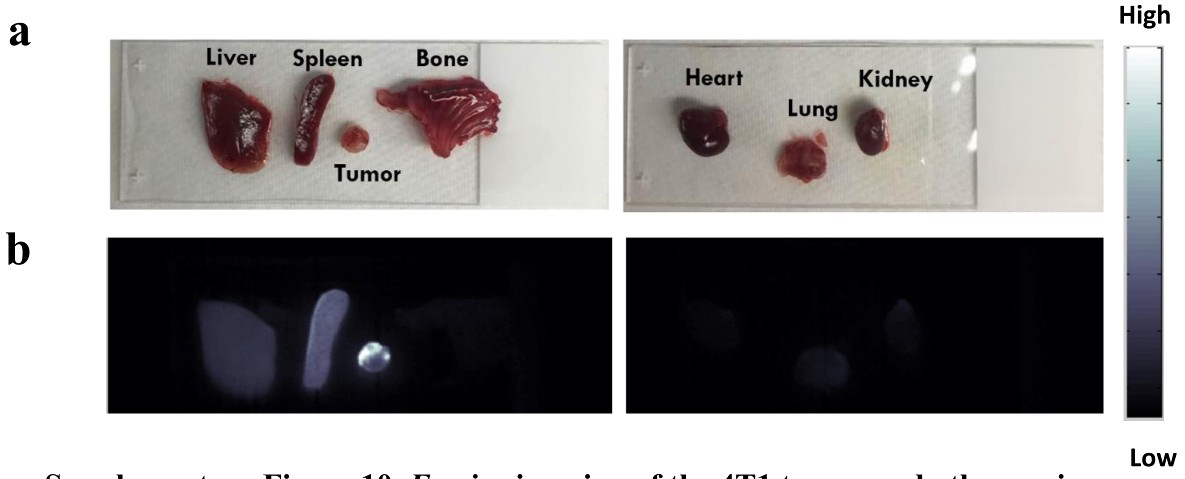

Supplementary Figure 10. ***Ex vivo*** **imaging of the 4T1 tumor and other major organs after post injection of p-FE. a,** Photos of the 4T1 tumor and other major organs and **b,** their corresponding *ex vivo* NIR-II fluorescence images.

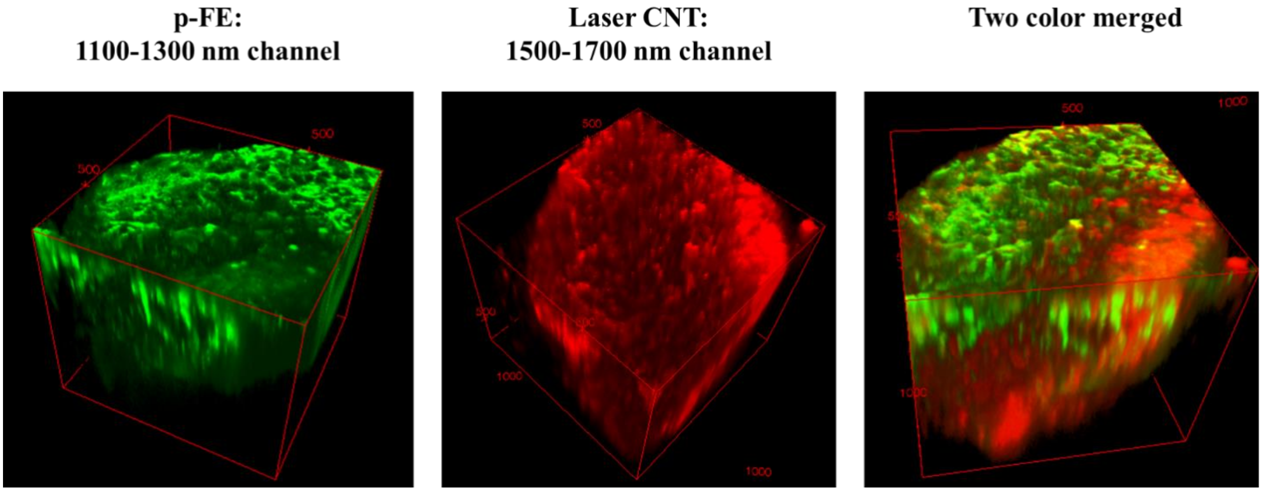

Supplementary Figure 11. ***Ex vivo*** **confocal imaging of the whole tumor using two colors in the NIR-II window.** 740 µm * 740 µm * 640 µm scanned area, step size: 2 µm in x and y, 3 µm in z. 740 µm * 740 µm * 640 µm area, step size: 2 µm along *x* and *y* directions, 5.4 µm along *z* direction. Laser power ~ 30 mW, PMT voltage: 500 V for p-FE channel and 600 V for laser CNT channel, scanning speed 15 min/frame. Pin hole: 150 µm for p-FE and 300 µm for laser CNT channel. Wavelength range: 1100 - 1300 nm for p-FE channel and 1500 - 1700 nm for laser CNT channel.

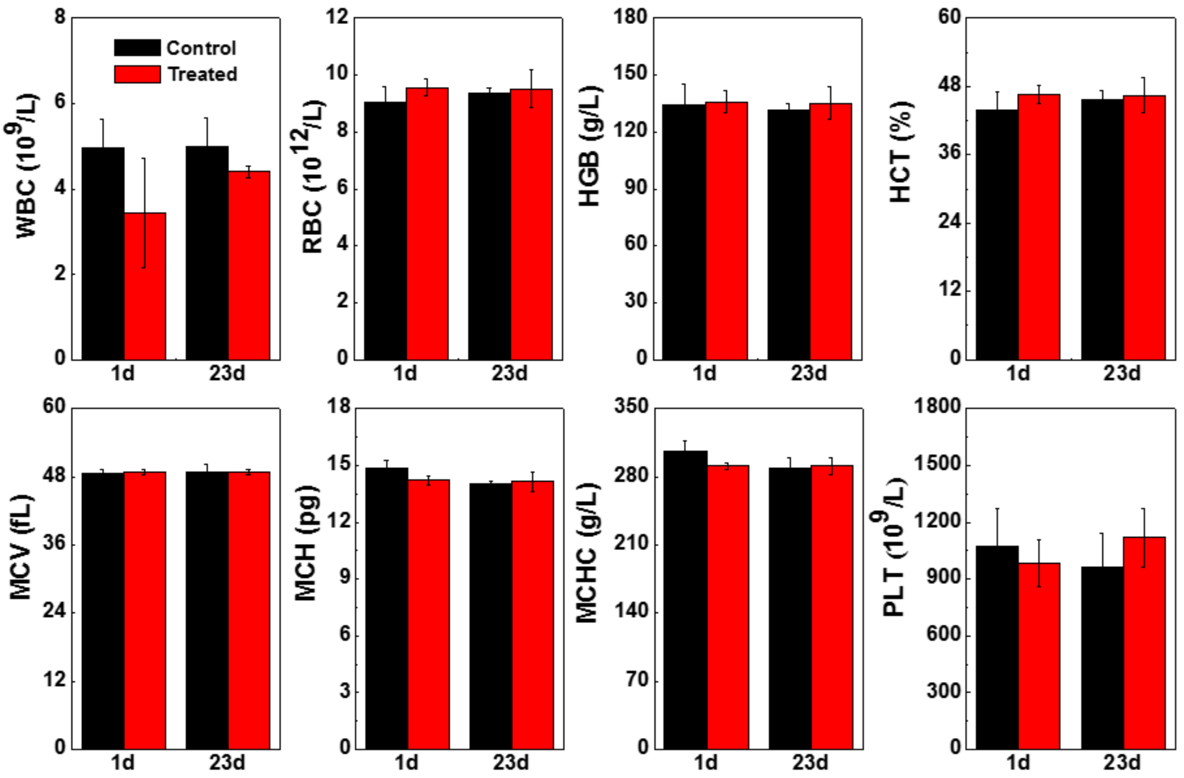

Supplementary Figure 12. Hematology analysis from healthy and p-FE treated mice performed at 1 and 23 days p.i (*n*=6 mice/group). The indicators include white blood cell (WBC), red blood cell (RBC), hemoglobin (HGB), platelet (PLT), mean corpuscular volume (MCV), mean corpuscular hemoglobin (MCH), mean corpuscular hemoglobin concentration (MCHC), hematocrit (HCT). No significant difference was found between control and p-FE treated groups. Error bars correspond to standard deviation.

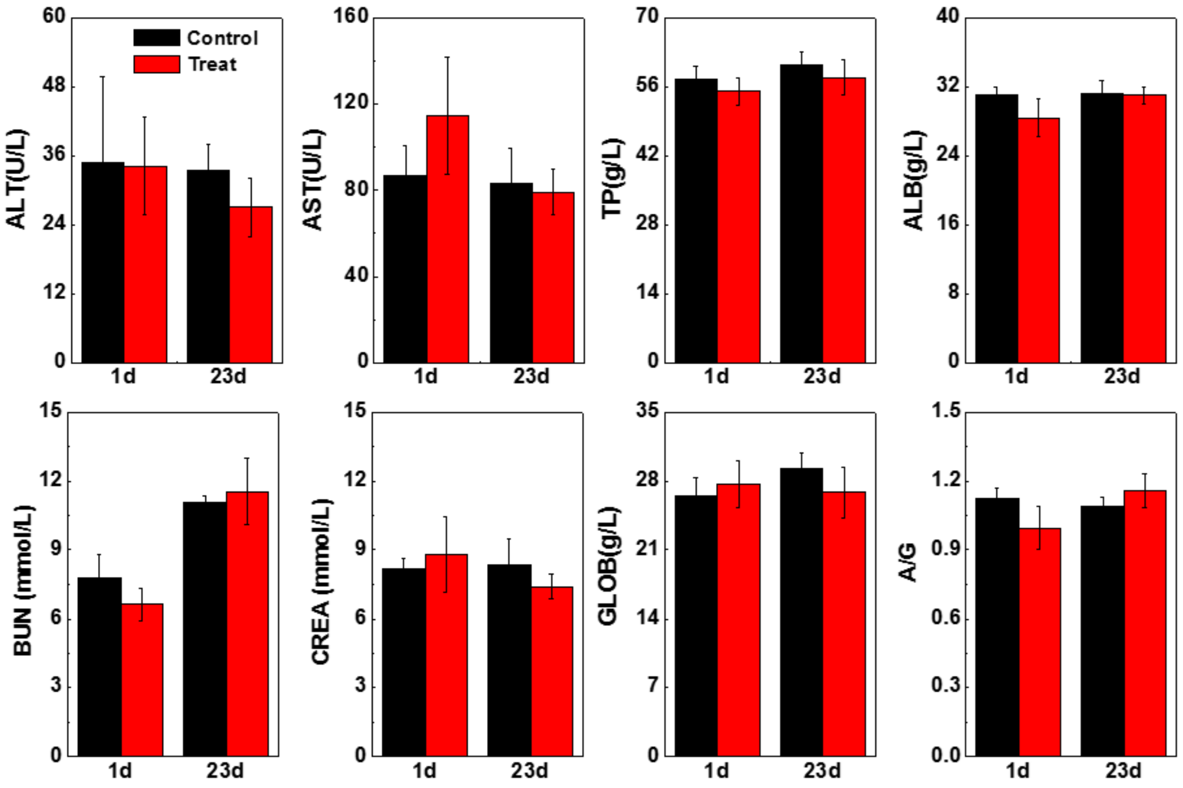

Supplementary Figure 13. Serum biochemistry analysis from healthy and p-FE treated mice carried at 1 day and 23 days p.i. (*n*=6 mice/group). The indicators include aspartate transaminase (AST), alanine transaminase (ALT), creatinine (CREA), blood urea nitrogen (BUN), albumin (ALB), total protein (TP), globulin (GLOB), albumin/globulin (A/G). No significant difference was found between control and p-FE treated groups. Error bars correspond to standard deviation.

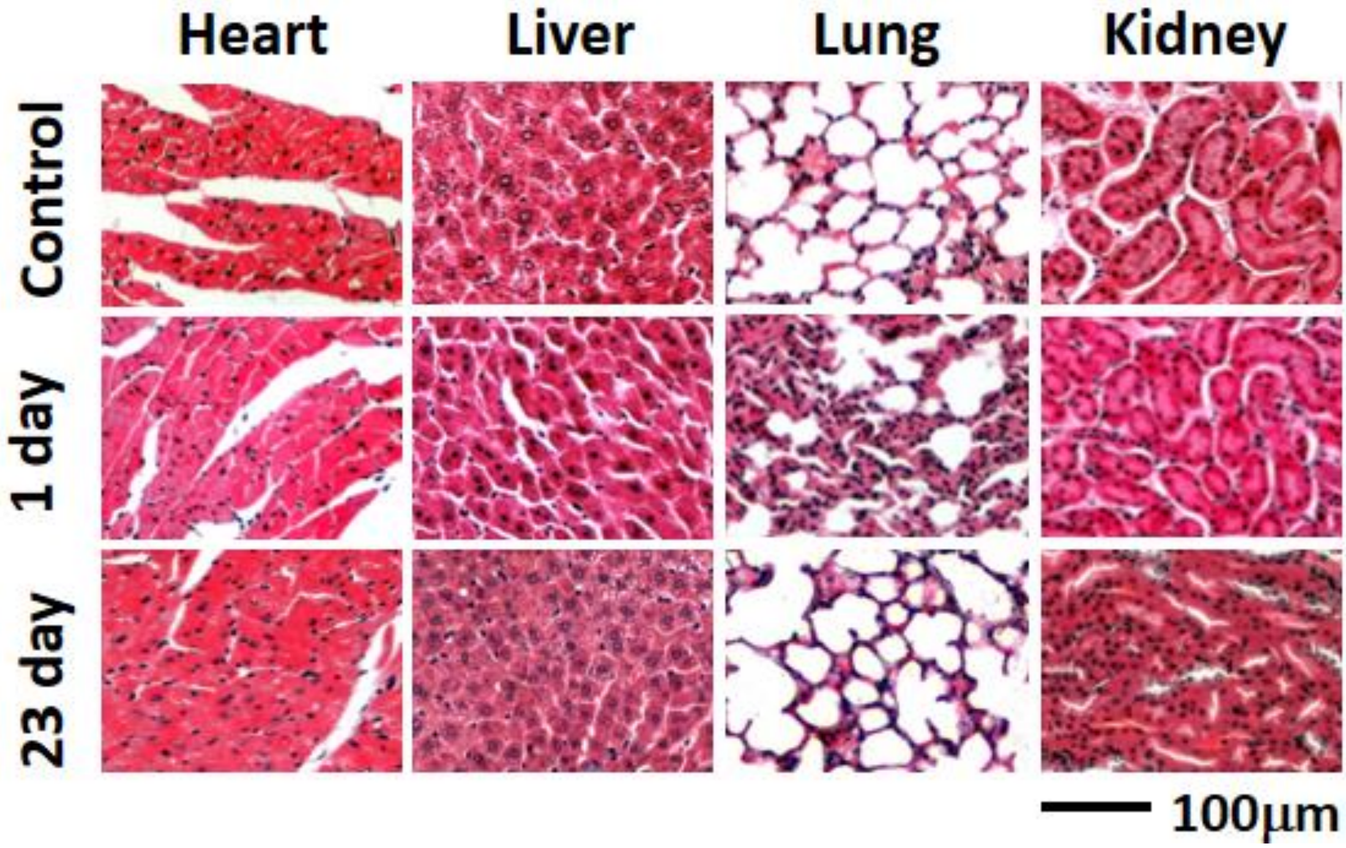

Supplementary Figure 14. Pathology images of healthy and p-FE treated mice at 1 and 23 days p.i.. All the main organs were processed according to a routine H&E staining procedure.

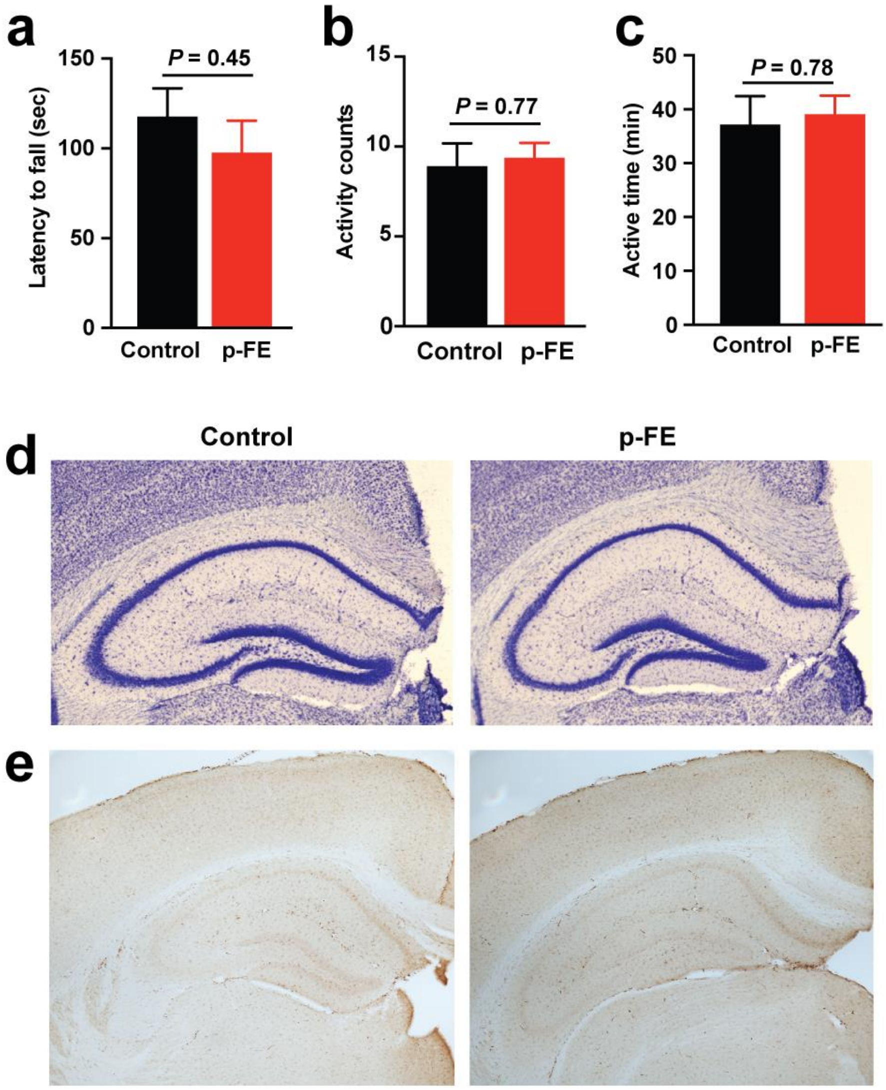

Supplementary Figure 15. Evaluation of neurobehavior and neurotoxicity of p-FE (*n* = 3 mice/group). a, Motor coordination assessed by RotaRod of control and of p-FE treated mice 5 days post injection. **b, c,** Locomotion assessed by SamrtCages. **d,** Morphologically, minimal tissue or cellular changes are observed in p-FE treated mice by Nissl (cresyl violet) stain. **e,** No signs of neuroinflammation in p-FE treated mice. Error bars correspond to standard deviation.
