# Supplementary material for: A bright organic NIR-II nanofluorophore for three-dimensional imaging into biological tissues
Source: Nat Commun. 2018 Mar 21;9:1171. doi: 10.1038/s41467-018-03505-4 (PMC5862886; doi:10.1038/s41467-018-03505-4)
Supplement: Supplementary file 2 — Description of Additional Supplementary Files(DOCX 14 kb) [file 41467_2018_3505_MOESM2_ESM.docx]

**Description of Additional Supplementary Files**

File Name: Supplementary Movie 1

Description: Real-time, beyond 30 FPS imaging of blood flow.

File Name: Supplementary Movie 2

Description: 3D reconstructed image animation.

File Name: Supplementary Movie 3

Description: Layer-by-layer image animation.

File Name: Supplementary Movie 4

Description: 3D reconstructed two-color image animation.
